# A machine learning model combining ultrasound features and serological markers predicts gallbladder polyp malignancy: A retrospective cohort study

**DOI:** 10.1097/MD.0000000000044371

**Published:** 2025-09-12

**Authors:** Yan Yang, Haibin Tu, Youguo Lin, Jianting Wei

**Affiliations:** a Preventive Treatment of Disease in Traditional Chinese Medicine, The Affiliated People’s Hospital of Fujian University of Traditional Chinese Medicine, Fuzhou, Fujian, China; b Department of Ultrasound, Mengchao Hepatobiliary Hospital of Fujian Medical University, Fuzhou, Fujian, China.

**Keywords:** gallbladder cancer, hematological markers, machine learning, prediction, ultrasound

## Abstract

Differentiating benign from malignant gallbladder polyps (GBPs) is critical for clinical decisions. Pathological biopsy, the gold standard, requires cholecystectomy, underscoring the need for noninvasive alternatives. This retrospective study included 202 patients (50 malignant, 152 benign) who underwent cholecystectomy (2018–2024) at Fujian Provincial Hospital. Ultrasound features (polyp diameter, stalk presence), serological markers (neutrophil-to-lymphocyte ratio [NLR], CA19-9), and demographics (age, sex, body mass index, waist-to-hip ratio, comorbidities, alcohol history) were analyzed. Patients were split into training (70%) and validation (30%) sets. Ten machine learning (ML) algorithms were trained; the model with the highest area under the receiver operating characteristic curve (AUC) was selected. Shapley additive explanations (SHAP) identified key predictors. Models were categorized as clinical (ultrasound + age), hematological (NLR + CA19-9), and combined (all 5 variables). ROC, precision-recall, calibration, and decision curve analysis curves were generated. A web-based calculator was developed. The Extra Trees model achieved the highest AUC (0.97 in training, 0.93 in validation). SHAP analysis highlighted polyp diameter, sessile morphology, NLR, age, and CA19-9 as top predictors. The combined model outperformed clinical (AUC 0.89) and hematological (AUC 0.68) models, with balanced sensitivity (66%–54%), specificity (94–93%), and accuracy (87%–83%). This ML model integrating ultrasound and serological markers accurately predicts GBP malignancy. The web-based calculator facilitates clinical adoption, potentially reducing unnecessary surgeries.

## 1. Introduction

Gallbladder polyps (GBPs) are frequently detected during abdominal ultrasonography, with a reported prevalence ranging from 0.3% to 12% in the general population.^[1,2]^ While the majority of GBPs are benign, a clinically significant proportion represents premalignant or malignant lesions.^[3]^ Accurate differentiation between benign and malignant GBPs is paramount for guiding appropriate clinical management, as early detection and surgical resection of malignant lesions are essential for improving patient prognosis.^[4]^

Ultrasound imaging plays a crucial role in the initial assessment of GBPs, providing vital information such as polyp size, number, morphology, and the presence of gallstones or gallbladder wall irregularities.^[5,6]^ Studies have indicated that specific ultrasound features, including polyp diameter exceeding 10 mm, sessile morphology, and single polyp presentation, are associated with an elevated risk of malignancy.^[7,8]^ However, the diagnostic accuracy of ultrasound alone remains limited, as overlapping features between benign and malignant polyps often lead to diagnostic ambiguity.^[9,10]^ Conversely, serological markers such as carbohydrate antigen 19-9 (CA19-9),^[11]^ carcinoembryonic antigen (CEA),^[12]^ and neutrophil-to-lymphocyte ratio (NLR)^[13]^ have been investigated as potential indicators of gallbladder malignancy, elevated levels of these markers have been correlated with malignant transformation, yet their independent predictive value is insufficient for clinical decision-making. Despite the complementary nature of ultrasound and serological markers, limited research has integrated these modalities to enhance the prediction of GBP malignancy.

Although both ultrasound features and serological markers offer valuable insights, their independent application has limitations. Notably, there is a scarcity of studies comprehensively integrating these 2 modalities for GBP malignancy prediction, representing a significant gap in the current diagnostic approach. The challenge lies in effectively combining these disparate data types to improve diagnostic accuracy.

Machine learning (ML) provides a robust solution to this challenge.^[14]^ ML algorithms are specifically designed to manage complex interactions and nonlinear relationships among multiple variables, rendering them ideally suited for integrating diverse data sources.^[15]^ In oncology, ML has demonstrated considerable success in enhancing diagnostic accuracy, predicting treatment response, and identifying prognostic factors across various cancer types. For instance, ML models incorporating radiomic features from CT scans have been utilized to predict lymph node metastasis in lung cancer.^[16]^ In breast cancer, ML algorithms integrating genomic data and clinical parameters have improved the prediction of recurrence risk.^[17]^ Furthermore, ML has been applied to differentiate benign from malignant liver lesions using a combination of imaging and serological data, achieving high diagnostic performance.^[18,19]^

Inspired by these advancements, we hypothesized that an ML model integrating both ultrasound features and serological biomarkers could significantly improve the noninvasive prediction of malignancy in GBPs. By leveraging the capacity of ML to analyze complex interactions between these data types, we aimed to develop a more accurate and reliable diagnostic tool. This study, therefore, sought to develop and validate such a model, potentially transforming the clinical management of GBPs by enabling more informed decision-making, reducing unnecessary cholecystectomies, and facilitating earlier detection of malignant lesions.

## 2. Methods

### 2.1. Study design and population

**Figure 1.**
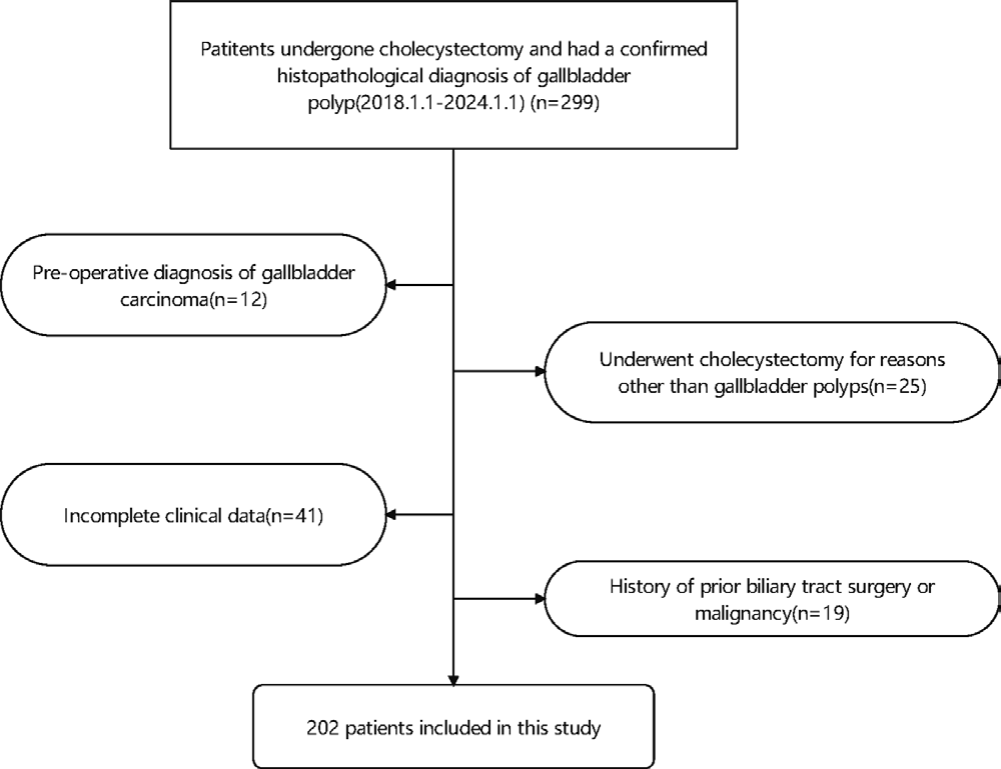
Patient inclusion flow.

This study employed a retrospective cohort design, reviewing data from patients who underwent cholecystectomy at Fujian Provincial People’s Hospital between January 1, 2018, and January 1, 2024. The study protocol was approved by the Institutional Review Board of Fujian Provincial People’s Hospital and conducted in accordance with the Declaration of Helsinki. Patients were included if they had undergone cholecystectomy and had a confirmed histopathological diagnosis of GBPs. Histopathology served as the reference standard for defining polyp malignancy status. Exclusion criteria were patients with a preoperative diagnosis of gallbladder carcinoma; patients who underwent cholecystectomy for reasons other than GBPs (e.g., cholecystitis, biliary dyskinesia without polyps); patients with incomplete clinical data, defined as missing ultrasound reports, serological marker results, or histopathology reports; and patients with a history of prior biliary tract surgery or malignancy. The patient inclusion flow was showed in Figure 1.

### 2.2. Data collection

Data were retrospectively extracted from electronic medical records, radiology information systems for ultrasound reports, and laboratory information systems for serological marker results. The following variables were collected for each patient:

#### 2.2.1. Ultrasound features

All examinations were performed using high-resolution ultrasound systems, including the Mindray Resona 7S/8S (Mindray Bio-Medical Electronics Co. Ltd, Shenzhen, China) and the Philips EPIQ 5 (Philips Healthcare, Bothell), equipped with a C5-2 transducer (Philips Healthcare), under standardized protocols. Two board-certified sonographers independently interpreted the findings through consensus-based evaluation. In cases of diagnostic discrepancy (observed in 3.8% of examinations), a third senior sonographer (with > 15 years’ experience) conducted blinded reassessment to achieve definitive interpretation. Finalized parameters were prospectively documented in radiology reports for subsequent analysis.

- Polyp diameter (mm): Maximum diameter of the largest polyp, measured in millimeters.
- Polyp number: Total number of polyps identified in the gallbladder, categorized as “single” or “multiple.”
- Polyp stalk: Presence or absence of a stalk, categorized as “pedunculated” (presence of a stalk) or “sessile” (absence of a stalk). In cases of pedunculated polyps, stalk thickness was not routinely measured and thus not included.
- Polyp location: Anatomical location of the polyp within the gallbladder, categorized as “bottom,” “body,” or “neck.”
- Polyp surface Characteristics: Described as “smooth” or “coarse” based on the ultrasound report.
- Echo homogeneity: Polyp echogenicity was categorized as “homogeneous” or “heterogeneous.”
- Hyperechoic foci: Presence or absence of hyperechoic foci within the polyp, documented as “yes” or “no.”
- Gallbladder stones: Presence or absence of gallstones within the gallbladder, documented as “yes” or “no.”
- Gallbladder wall roughness: Presence or absence of gallbladder wall roughness, described in reports as irregular or thickened gallbladder wall contour, documented as “yes” or “no.” This was distinct from gallbladder wall thickness, which was not consistently reported and therefore not included.

#### 2.2.2. Serological markers

Preoperative serum samples were collected within 1 week prior to cholecystectomy. The following hematological markers were recorded, with units of measurement specified:

- Alanine aminotransferase (ALT, U/L)
- Aspartate aminotransferase (AST, U/L)
- Total bilirubin (TBIL, μmol/L)
- NLR: Calculated as absolute neutrophil count divided by absolute lymphocyte count
- Carbohydrate antigen 19-9 (CA19-9, U/mL)
- Carcinoembryonic antigen (CEA, ng/mL)
- Alkaline phosphatase (ALP, U/L)
- Adenosine deaminase (ADA, IU/L)

#### 2.2.3. Demographic and clinical data

The following demographic and clinical variables were collected:

- Age (years): Age at the time of cholecystectomy.
- Sex: Categorized as “male” or “female.”
- Body mass index (BMI, kg/m^2^): Calculated as weight in kilograms divided by height in meters squared.
- Waist-to-hip ratio: Measured at the time of admission, calculated as waist circumference divided by hip circumference, and categorized as “normal” or “fatty.”
- Comorbidities: Presence or absence of hypertension, diabetes, hyperlipidemia, and viral hepatitis, documented as “yes” or “no.”
- Alcohol History: History of alcohol consumption, documented as “yes” or “no.”

### 2.3. Data preprocessing

Prior to model development, data preprocessing steps were undertaken. Missing values, present in <5% of the dataset and primarily in CEA and ALP measurements, were imputed using median imputation, as these markers were not expected to be highly skewed. Outliers were assessed using boxplots and defined as values exceeding 1.5 times the interquartile range above the 75th percentile or below the 25th percentile. No outliers were removed as clinically implausible, but extreme values were winsorized to the 99th percentile to mitigate undue influence on model training. Continuous variables were standardized using z-score normalization to ensure features were on a comparable scale, improving the performance and convergence of certain ML algorithms.

### 2.4. ML model development

The dataset was randomly split into a training set (70%) and a validation set (30%) using a fixed random seed (seed = 123456) to ensure reproducibility. Ten ML algorithms were employed for model development using the scikit-learn library in Python (3.6.1). The algorithms included: Logistic Regression, Random Forest, Gradient Boosting, Support Vector Classifier (SVC) with a radial basis function kernel, Decision Tree, K-Nearest Neighbors, Naive Bayes (Gaussian Naive Bayes), LightGBM, Extra Trees, and AdaBoost.

Hyperparameter tuning for each algorithm was performed using a grid search approach with 10-fold cross-validation on the training set to optimize model performance and prevent overfitting. Ten-fold cross-validation was implemented to robustly estimate the performance of each model on unseen data within the training set. In each fold, the training data was further divided into 9-folds for training and 1-fold for validation. The average area under the receiver operating characteristic curve (AUC) across the 10-folds was used to evaluate each algorithm’s performance.

Model selection was based on the highest average AUC achieved during the 10-fold cross-validation in the training set. The Extra Trees algorithm demonstrated the highest AUC and was selected for further analysis and evaluation on the validation set.

Feature importance analysis was conducted using Shapley additive explanations (SHAP) values, SHAP values provide a unified measure of feature importance by quantifying the contribution of each feature to the prediction of individual instances. The top 10 most important variables, ranked by mean absolute SHAP value, were visualized to understand their relative influence on the model’s predictions.

To explore model parsimony, we iteratively built models with varying numbers of top-ranked features, starting with the single most important feature and incrementally adding features based on their SHAP ranking. The AUC was calculated for each model configuration using 10-fold cross-validation on the training set to identify the optimal number of variables that maximized performance while maintaining model simplicity.

Based on the variables included, 3 model categories were defined:

- Clinical model: Included polyp diameter, polyp stalk (sessile/pedunculated), and patient age – variables readily available from routine clinical assessment and basic ultrasound.
- Hematological model: Included NLR and carbohydrate antigen 19-9 (CA19-9) – serological markers with potential relevance to inflammation and malignancy.
- Combined model: Included all collected variables, representing the integration of clinical, ultrasound, and hematological data.

### 2.5. Model evaluation

The performance of the selected Extra Trees model and the 3 defined model categories (Clinical, Hematological, and Combined) were evaluated on the independent validation set. Performance metrics included: AUC, sensitivity, specificity, positive predictive value (PPV), negative predictive value (NPV), accuracy. Receiver operating characteristic (ROC) curves and precision-recall (PR) curves were plotted to visualize the trade-off between sensitivity and specificity, and precision and recall, respectively. Calibration curves were generated to assess the reliability of the predicted probabilities, using isotonic regression for calibration. Decision curve analysis (DCA) was performed to evaluate the clinical utility of the models by quantifying the net benefit across a range of clinically relevant risk thresholds.

### 2.6. Web-based calculator development

To facilitate clinical translation, a user-friendly web-based calculator was developed using specify web framework. The calculator is hosted at https://gallbladder-kwljafh4ile9dlb9qu9y46.streamlit.app/.” The calculator allows clinicians to input patient clinical characteristics, ultrasound features, and serological marker values, and obtain a predicted probability of gallbladder polyp malignancy based on the combined model. The calculator interface is designed for ease of use and rapid risk assessment in a clinical setting.

### 2.7. Statistical analysis

Statistical analyses were performed using Python (version 3.6.1; Pytho n Software Foundation, Wilmington) with the libraries scikit-learn, pandas, numpy, matplotlib, seaborn, and statsmodels). Continuous variables are presented as mean ± standard deviation (SD) or median (interquartile range [IQR]) depending on normality, assessed using the Shapiro–Wilk validation. Categorical variables are presented as frequencies and percentages. Differences between the training and validation sets for continuous variables were assessed using the independent samples t-validation or Mann-Whitney U validation as appropriate, and for categorical variables using the chi-square validation. A 2-sided *P*-value < .05 was considered statistically significant.

## 3. Results

### 3.1. Basic information of all patients

This study included a total of 202 patients, of whom 50 were diagnosed with gallbladder cancer. The cohort was divided into a training set (n = 142, including 35 with gallbladder cancer) and a validation set (n = 60, including 15 with gallbladder cancer). Of the entire cohort, 110 patients (54.5%) were male, and the mean age was 59.9 ± 15.89 years. Additional baseline characteristics are presented in Table 1. Crucially, the training and validation sets were well-balanced, with no statistically significant differences observed between the groups for any of the measured variables (all *P* > .05). The information in the training set is shown in Table S1 (Supplemental Digital Content, https://links.lww.com/MD/P888).

**Table 1:** Basic information of all patients.

Variable	Total	Training	Validation	Statistic	*P*-value
Gallbladder polyps	152 (75.2%)	107 (75.4%)	45 (75%)	0	1
Gallbladder cancer	50 (24.8%)	35 (24.6%)	15 (25%)		
Female	92 (45.5%)	67 (47.2%)	25 (41.7%)	0.32	.57
Male	110 (54.5%)	75 (52.8%)	35 (58.3%)		
Stalk (sessile)	112 (55.4%)	85 (59.9%)	27 (45%)	3.19	.07
Stalk (pedunculated)	90 (44.6%)	57 (40.1%)	33 (55%)		
Waist_Hip_Ratio (Normal)	95 (47%)	67 (47.2%)	28 (46.7%)	0	1
Waist_Hip_Ratio (fatty)	107 (53%)	75 (52.8%)	32 (53.3%)		
Hypertension (no)	152 (75.2%)	104 (73.2%)	48 (80%)	0.7	.4
Hypertension (yes)	50 (24.8%)	38 (26.8%)	12 (20%)		
Diabetes (no)	173 (85.6%)	124 (87.3%)	49 (81.7%)	0.69	.41
Diabetes (yes)	29 (14.4%)	18 (12.7%)	11 (18.3%)		
Hyperlipidemia (no)	157 (77.7%)	115 (81%)	42 (70%)	2.34	.13
Hyperlipidemia (yes)	45 (22.3%)	27 (19%)	18 (30%)		
Alcohol history (no)	192 (95%)	134 (94.4%)	58 (96.7%)	0.11	.74
Alcohol history (yes)	10 (5%)	8 (5.6%)	2 (3.3%)		
Polyp_Location (bottom)	79 (39.1%)	54 (38%)	25 (41.7%)	0.42	.81
Polyp_Location (body)	74 (36.6%)	54 (38%)	20 (33.3%)		
Polyp_Location (neck)	49 (24.3%)	34 (23.9%)	15 (25%)		
Polyp_Number (single)	100 (49.5%)	68 (47.9%)	32 (53.3%)	0.31	.58
Polyp_Number (multiple)	102 (50.5%)	74 (52.1%)	28 (46.7%)		
Echo_Homogeneity	75 (37.1%)	52 (36.6%)	23 (38.3%)	0.01	.94
Echo_Heterogeneous	127 (62.9%)	90 (63.4%)	37 (61.7%)		
Polyp Surface Smoothness	170 (84.2%)	122 (85.9%)	48 (80%)	0.71	.4
Polyp Surface Coarse	32 (15.8%)	20 (14.1%)	12 (20%)		
Hyperechoic_Foci (no)	101 (50%)	77 (54.2%)	24 (40%)	2.87	.09
Hyperechoic_Foci (yes)	101 (50%)	65 (45.8%)	36 (60%)		
Gallstones (no)	61 (30.2%)	43 (30.3%)	18 (30%)	0	1
Gallstones (yes)	141 (69.8%)	99 (69.7%)	42 (70%)		
Gallbladder_Smooth	155 (76.7%)	108 (76.1%)	47 (78.3%)	0.03	.87
Gallbladder_Roughness	47 (23.3%)	34 (23.9%)	13 (21.7%)		
Viral_Hepatitis (no)	81 (40.1%)	59 (41.5%)	22 (36.7%)	0.24	.62
Viral_Hepatitis (yes)	121 (59.9%)	83 (58.5%)	38 (63.3%)		
Age (yr)	59.9 ± 15.89	59.45 ± 15.95	60.97 ± 15.84	0.44	.5
Diameter (mm)	13.99 ± 3.32	13.75 ± 3.43	14.55 ± 2.99	2.51	.11
NLR	2.97 ± 1.82	3.03 ± 1.82	2.82 ± 1.83	0.66	.42
BMI	22.1 ± 4.01	22.1 ± 3.96	22.09 ± 4.18	0	.99
TBIL (g/dL)	16.9 ± 8.71	16.72 ± 8.79	17.33 ± 8.6	0.29	.59
ADA (IU/L)	12.1 ± 8.03	12.58 ± 8.14	10.98 ± 7.71	2.14	.14
CEA (ng/mL)	4.07 ± 3.87	4.21 ± 3.96	3.74 ± 3.66	0.78	.38
CA19-9 (U/mL)	24.24 ± 20.19	23.54 ± 19.9	25.89 ± 20.94	0.97	.33
ALP (IU/L)	104.67 ± 50.22	106.18 ± 52.89	101.1 ± 43.45	0	.96
AST (IU/L)	29.74 ± 16.07	28.88 ± 15.58	31.78 ± 17.15	1.56	.21
ALT (IU/L)	34.82 ± 21.73	33.48 ± 21.56	37.98 ± 21.99	2.71	.1

ADA = adenosine deaminase, ALP = alkaline phosphatase, ALT = alanine aminotransferase, AST = aspartate aminotransferase, BMI = body mass index, CA199 = carbohydrate antigen 19-9, CEA = carcinoembryonic antigen, NLR = neutrophil-to-lymphocyte ratio, TBIL = total bilirubin.

### 3.2. Comparison of predictive performance of different ML models

Table 2 presents a comprehensive comparison of the predictive performance of various ML models using multiple evaluation metrics, including mean AUC, 95% confidence intervals for AUC, mean accuracy, sensitivity, specificity, PPV, and NPV. Among the models evaluated, the Extra Trees classifier demonstrated the highest predictive performance, with a mean AUC of 0.97 (95% CI: 0.94–0.99), significantly outperforming other models. Additionally, the Extra Trees model achieved the highest mean accuracy (0.95), sensitivity (0.95), specificity (0.94), PPV (0.92), and NPV (0.92), indicating its robust ability to discriminate between positive and negative cases. Given its superior performance across all metrics, the Extra Trees model was selected for further analysis in this study.

**Table 2:** Comparison of predictive performance of different machine learning.

Model	Mean AUC	AUC 95% CI lower	AUC 95% CI upper	Mean accuracy	Sensitivity	Specificity	PPV	NPV
Logistic Regression	0.85	0.75	0.9	0.83	0.92	0.75	0.9	0.8
Random Forest	0.9	0.81	0.99	0.91	0.95	0.87	0.92	0.88
Gradient Boosting	0.88	0.8	0.99	0.89	0.88	0.91	0.89	0.9
SVC	0.76	0.69	0.85	0.78	0.81	0.82	0.79	0.84
Decision Tree	0.89	0.84	0.94	0.91	0.9	0.85	0.91	0.86
K-Nearest Neighbors	0.88	0.83	0.94	0.83	0.54	0.95	0.94	0.86
Naive Bayes	0.85	0.81	0.88	0.82	0.7	0.79	0.85	0.83
LightGBM	0.8	0.76	0.85	0.76	0.85	0.92	0.72	0.77
Extra Trees	0.97	0.94	0.99	0.95	0.95	0.94	0.92	0.92
AdaBoost	0.86	0.84	0.89	0.83	0.7	0.95	0.72	0.91

AUC = area under the curve, CI = confidence interval, NPV = negative predictive value, PPV = positive predictive value, SVC = support vector classifier.

### 3.3. Selection of the optimal number of variables

**Figure 2.**
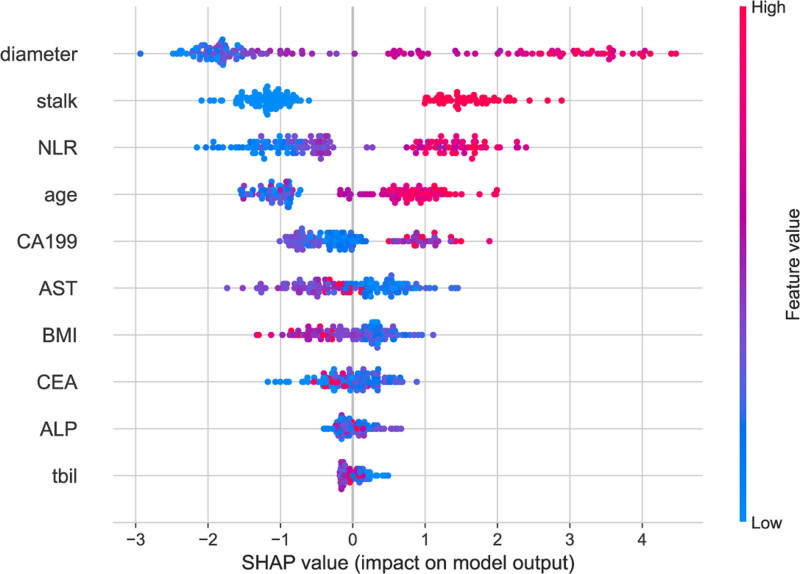
SHAP summary plot of feature importance for gallbladder polyp malignancy prediction. This plot displays the distribution of SHAP values for the top 10 features in the Extra Trees model. Each point represents 1 patient; the *x*-axis shows SHAP value magnitude, and the color indicates the corresponding feature value (red = high, blue = low). Features are ranked by their mean absolute SHAP value. SHAP = Shapley additive explanations.

**Figure 3.**
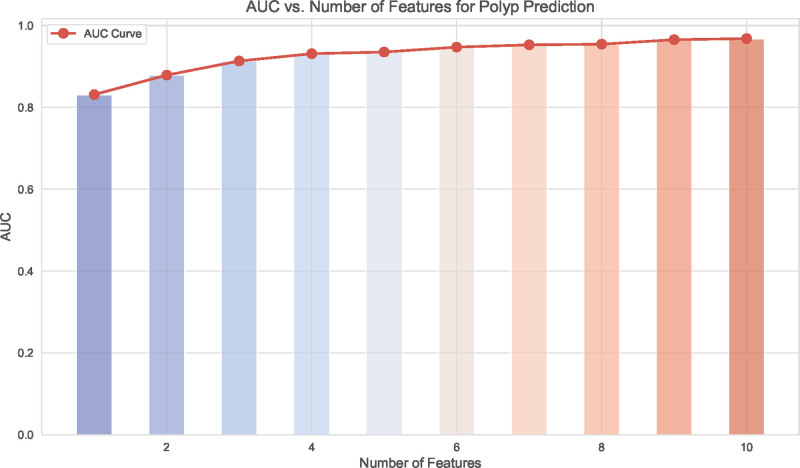
AUC vs number of features for polyp prediction. The bar plot shows the relationship between the number of features used in the model and the corresponding AUC. The red curve illustrates the AUC trend as the number of included features increases. AUC = area under the receiver operating characteristic curve.

After selecting the Extra Trees model as the predictive algorithm, we ranked the variables based on their importance and visualized the results using SHAP, as illustrated in Figure 2. The top 10 variables identified were: diameter, stalk, NLR, age, CA19-9, AST, BMI, CEA, ALP, and TBIL. To adhere to the principle of model parsimony, we calculated the corresponding AUC values for models incorporating different numbers of variables, as shown in Figure 3. The results demonstrated that the AUC reached a high value when the model included 5 variables, and further inclusion of additional variables did not significantly improve the AUC. Therefore, for subsequent analyses, we constructed a fusion model incorporating the following 5 variables: diameter, stalk, NLR, age, and CA19-9.

### 3.4. Model classification and comparative analysis

**Figure 4.**
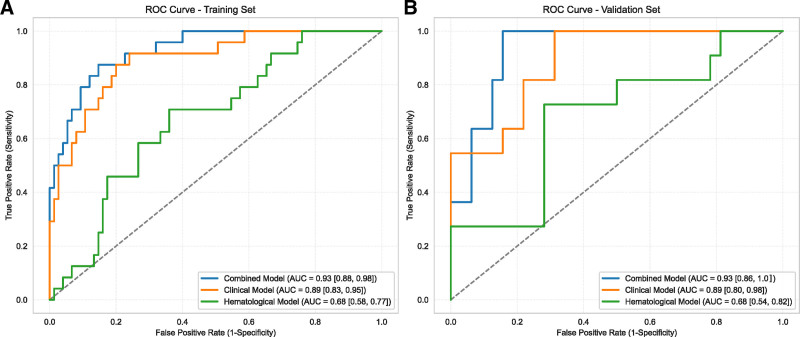
Receiver operating characteristic (ROC) curves of different models on the training and validation sets. ROC curves for the combined, clinical, and hematological models are shown for the training set (A) and validation set (B). The curves illustrate the diagnostic performance of each model.

**Figure 5.**
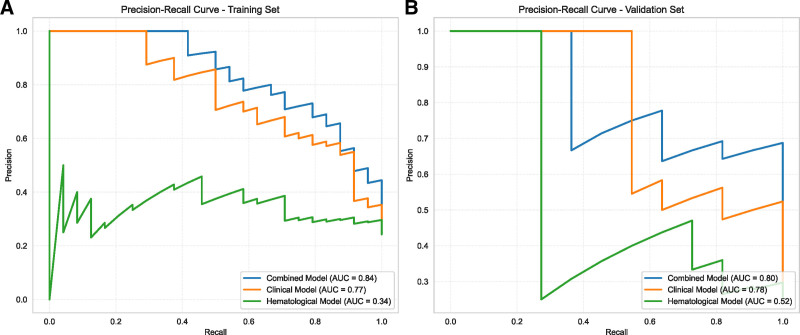
PR curves of the combined, clinical, and hematological models on the training and validation sets. PR curves for the 3 models are presented for the training set (A) and validation set (B), with recall on the *x*-axis and precision on the *y*-axis. PR = precision-recall.

Based on the selected variables, we categorized diameter, stalk, and age as the “Clinical Model.” CA19-9 and NLR were classified as the “Hematological Model.” We then compared the predictive performance of these 2 models, along with a “combined model” incorporating all 5 variables. ROC curves for each model are presented in Figure 4, and the PR curve is presented in Figure 5. Both the ROC and PR curves demonstrated the superior performance of the combined model. AUC values and performance metrics are summarized in Table 3. The combined model consistently outperformed the other 2 models, achieving the highest AUC values (0.93 for both training and validation sets) and demonstrating balanced sensitivity, specificity, and accuracy.

**Table 3:** Comparison of predictive performance of different models.

Dataset	Model	AUC	AUC 95% CI lower	AUC 95% CI upper	Sensitivity	Specificity	PPV	NPV	Accuracy
Train	Combined model	0.93	0.88	0.98	0.66	0.94	0.8	0.89	0.87
	Clinical model	0.89	0.83	0.95	0.54	0.93	0.72	0.86	0.83
	Hematological model	0.68	0.58	0.77	0	1	0	0.75	0.75
Validation	Combined model	0.93	0.86	1	0.54	0.93	0.75	0.85	0.83
	Clinical model	0.89	0.8	0.98	0.54	0.96	0.85	0.86	0.86
	Hematological model	0.68	0.54	0.82	0	1	0	0.74	0.74

AUC = area under the curve, CI = confidence interval, NPV = negative predictive value, PPV = positive predictive value.

### 3.5. Calibration curve

**Figure 6.**
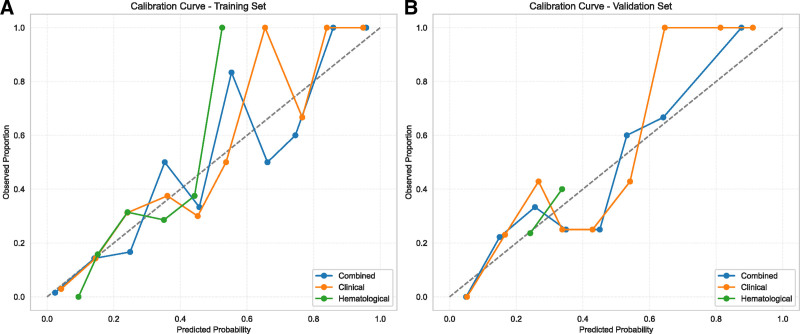
Calibration curves of the combined, clinical, and hematological models on the training and validation sets. Calibration plots are shown for the training set (A) and validation set (B), with predicted probability on the *x*-axis and observed frequency on the *y*-axis.

Figure 6 displays the calibration curves for the training and validation cohorts. In both datasets, the combined model exhibits the best calibration, with its curve tracking closest to the ideal 45-degree diagonal, indicating good agreement between predicted probabilities and observed proportions. The Clinical Model shows a slight tendency towards over-calibration in the higher probability ranges, particularly in the validation set. The Hematological Model demonstrates the least satisfactory calibration, deviating more noticeably from the ideal line in both datasets. These calibration plots visually confirm the superior calibration of the combined model compared to the other 2 models. Table 4 presents the calibration metrics for all models. The combined model demonstrated good calibration in both the training and validation sets. In the training set, the combined model had a Brier score of 0.09, an HL *P*-value of .25, a calibration slope of 1.03, and a calibration intercept of < 0.01. In the validation set, the combined model maintained good calibration, with a Brier score of 0.08, an HL *P*-value of .32, a calibration slope of 0.95, and a calibration intercept of 0.11. These values indicate that the combined model’s predicted probabilities are well-calibrated with the observed outcomes. The clinical and hematological models showed less optimal calibration, as detailed in Table 4.

**Table 4:** Calibration metrics and NRI/IDI of different models in different cohorts.

Dataset	Model	Brier score	HL *P*-value	Calibration slope	Calibration intercept	NRI	IDI
Train	Combined	0.09	.25	1.03	<0.01		
	Clinical	0.12	.2	1.16	-0.02	0.11	0.1
	Hematological	0.17	<.01	1.76	-0.17	0.62	0.41
Validation	Combined	0.08	.32	0.95	0.11		
	Clinical	0.15	.03	0.85	0.25	0.15	0.11
	Hematological	0.19	<.01	0.25	0.32	0.44	0.29

HL: Hosmer–Lemeshow; NRI: net reclassification improvement; IDI: integrated discrimination improvement.

### 3.6. DCA

**Figure 7.**
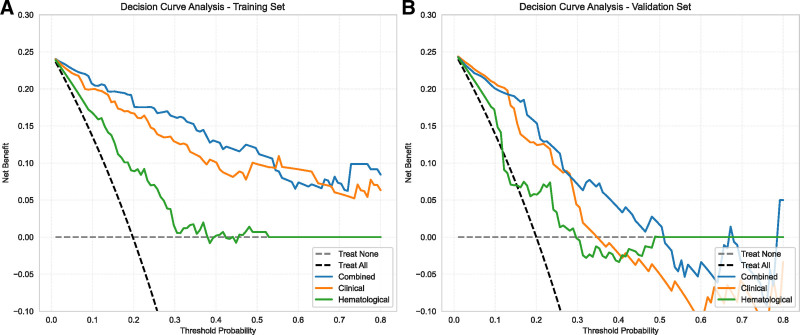
DCA of the combined, clinical, and hematological models on the training and validation sets. Decision curves are presented for training (A) and validation (B) datasets. The *x*-axis represents threshold probability, and the *y*-axis denotes net benefit. DCA = decision curve analysis.

Net reclassification improvement, and integrated discrimination improvement values comparing the clinical and hematological models to the combined model are also presented in Table 4. Combined model demonstrated superior performance across all other evaluation metrics. DCA; Figure 7 further evaluated the clinical utility of the models. The combined model exhibited the highest net benefit across a wide range of threshold probabilities in both the training and validation sets, indicating its superior clinical value compared to the clinical and hematological models, as well as the strategies of treating all or treating no patients.

### 3.7. Individualized prediction explanations using waterfall plot

**Figure 8.**
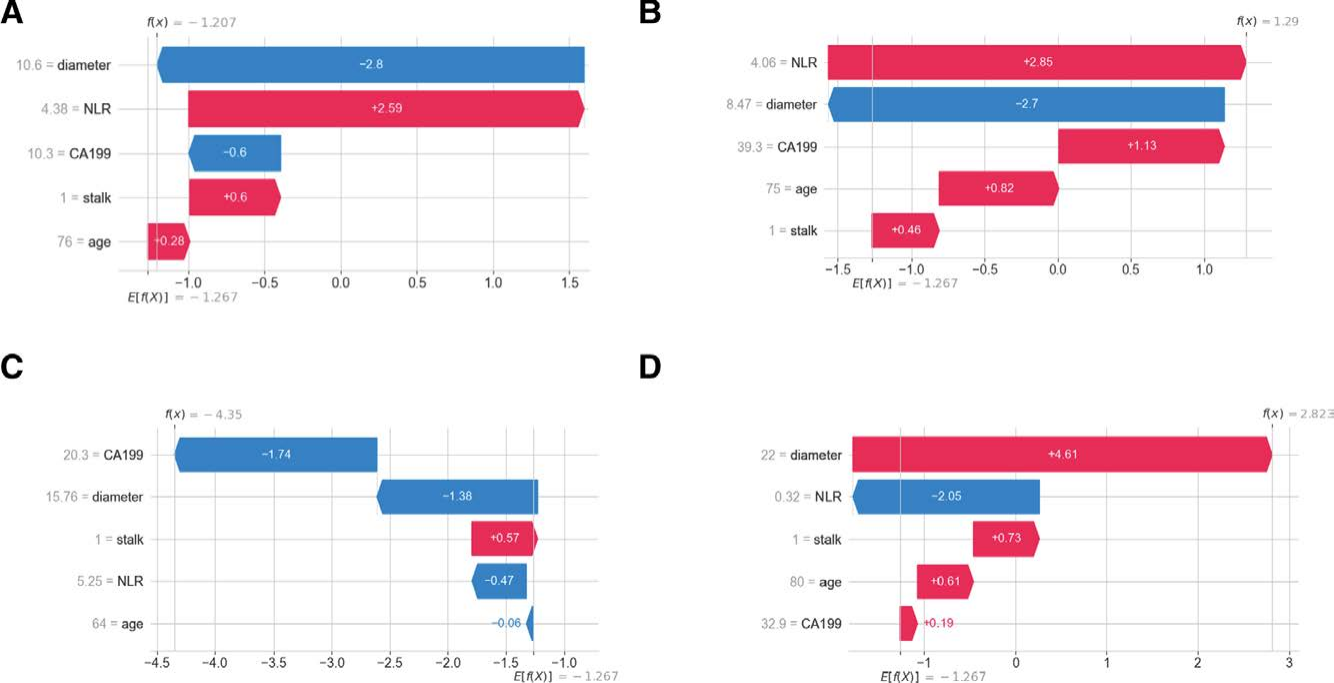
Individualized prediction explanations using waterfall plots. Waterfall plots illustrate feature contributions to prediction outputs in 4 representative cases. Red bars represent features increasing the prediction score, and blue bars represent features decreasing it. (A) A true negative case correctly classified as low risk, primarily due to a small polyp diameter. (B) A false positive case misclassified as high risk, largely influenced by an elevated neutrophil-to-lymphocyte ratio (NLR). (C) A false negative case incorrectly classified as benign, despite an elevated carbohydrate antigen 19-9 (CA19-9) level, owing to the influence of smaller polyp diameter. (D) A true positive case correctly identified as malignant, primarily driven by a significantly larger polyp diameter.

To provide deeper insight into the decision-making process of the Extra Trees model, we visualized individual predictions using SHAP force plots (Fig. 8). These plots reveal how specific feature values contribute to each prediction, shifting it from the average model output towards either malignancy or benignity. For instance, a true negative case (Fig. 8A) was correctly classified primarily due to a small polyp diameter, a well-established indicator of low risk. Conversely, a false positive prediction (Fig. 8B) was driven by a moderately elevated NLR, suggesting that systemic inflammation can, in some cases, outweigh the influence of favorable ultrasound characteristics. A false negative case (Fig. 8C) illustrates the complexity of the interplay; despite an elevated CA19-9, other features, such as a smaller diameter, led to an incorrect benign prediction. Finally, a true positive case (Fig. 8D) was correctly identified, largely due to a significantly larger polyp diameter, reinforcing the critical role of size in malignancy risk. These individual case analyses underscore the model’s ability to integrate diverse data types and highlight the nuanced interplay between clinical, ultrasound, and serological features in predicting gallbladder polyp malignancy. They also reveal potential areas for future refinement, such as incorporating additional biomarkers or imaging modalities to improve accuracy in cases where conflicting indicators are present.

### 3.8. Web-based calculator for gallbladder polyp malignancy prediction

**Figure 9.**
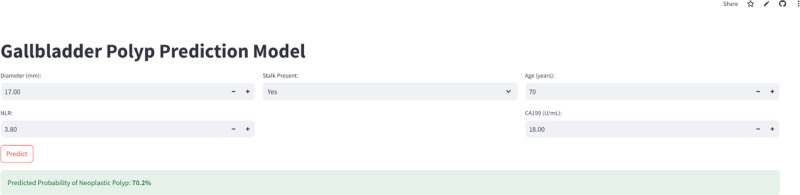
Web-based calculator for gallbladder polyp malignancy prediction. The screenshot shows the user interface of the developed web-based calculator, where clinicians can input variables to generate predicted malignancy probability.

To facilitate the clinical translation of our prediction model, we developed a user-friendly, web-based calculator (Fig. 9): https://gallbladder-kwljafh4ile9dlb9qu9y46.streamlit.app/. This tool allows clinicians to easily input the 5 key variables – polyp diameter, stalk presence, age, NLR, and CA19-9 – and obtain an immediate prediction of malignancy risk. The calculator, built upon the combined model, provides a readily accessible and practical means of implementing our research findings in a clinical setting. By providing a quantitative risk assessment, the calculator has the potential to aid in clinical decision-making, promoting more personalized management strategies for patients with GBPs. This represents a crucial step towards moving beyond subjective assessments and towards a more data-driven approach to GBP management.

## 4. Discussion

This retrospective study successfully developed and validated a noninvasive ML model for predicting gallbladder polyp malignancy, demonstrating excellent performance in our validation cohort. Our key finding is the robust predictive capability of a combined model integrating readily available clinical data, ultrasound features, and serological markers, outperforming models relying on single data modalities. The Extra Trees algorithm emerged as the optimal ML approach, achieving a high AUC of 0.97 in training and robust performance in validation. Furthermore, SHAP analysis provided valuable insights into the key predictors driving model performance, identifying polyp diameter, stalk morphology, NLR, age, and CA19-9 as the most influential variables. Finally, the development of a user-friendly web-based calculator represents a significant step towards clinical translation of our model.

Consistent with established literature, polyp diameter emerged as a dominant predictor of malignancy in our model. Larger polyp size has been repeatedly shown to correlate with increased risk of gallbladder cancer.^[20,21]^ This is biologically plausible as larger polyps are more likely to harbor dysplastic or malignant transformation due to prolonged growth and increased cellular turnover. Our model effectively leveraged this well-established clinical risk factor, further validating its clinical relevance.^[9]^

Sessile polyp morphology, indicated by the absence of a stalk (i.e., a wide-based or sessile polyp), was also identified as a significant predictor. Sessile polyps are known to have a higher malignant potential compared to pedunculated polyps, Wang research also found that when the base of the polyp widens, it is more likely to be malignant.^[22]^ Wang’s study also found that pedunculated polyps had a higher rate of malignancy compared to sessile polyps.^[23]^ This may be attributed to their broader attachment to the gallbladder wall, potentially facilitating deeper invasion and lymphatic spread in case of malignancy. The model’s ability to incorporate this morphological feature underscores its capacity to capture nuanced ultrasound characteristics beyond simple size.

Advanced age was another important predictor identified by SHAP analysis. Increasing age is a well-recognized risk factor for various cancers,^[24,25]^ including gallbladder cancer.^[26,27]^ This reflects the cumulative effect of genetic mutations, environmental exposures, and declining immune surveillance over time, increasing the likelihood of malignant transformation in GBPs as well.^[28,29]^

Elevated levels of CA19-9, a serological marker commonly associated with pancreatobiliary malignancies, also contributed significantly to the model’s predictive power.^[30]^ While CA19-9 is not recommended for routine screening of gallbladder cancer, its elevation can reflect underlying malignant processes and has been shown to correlate with advanced stage and poorer prognosis in gallbladder cancer.^[31]^ Our findings suggest that even within the context of GBPs, preoperative CA19-9 levels can provide valuable information regarding malignancy risk, enhancing the discriminatory ability of our model.

Finally, a higher NLR was identified as a significant predictor. NLR, a readily available marker of systemic inflammation, has gained increasing attention as a prognostic indicator in various cancers.^[13]^ Chronic inflammation is recognized as a key driver of carcinogenesis, and an elevated NLR, reflecting a pro-tumorigenic inflammatory microenvironment, may indicate a higher likelihood of malignancy within a GBP. The inclusion of NLR in our model highlights the potential of readily available systemic inflammatory markers to improve noninvasive risk stratification.^[32,33]^

Compared to previous studies focusing primarily on ultrasound features or clinical risk factors alone, our study’s novelty lies in the integrated approach, combining ultrasound morphology, serological markers, and clinical data within an ML framework. While some studies have explored radiomics or advanced imaging techniques for gallbladder lesion characterization,^[34]^ our model leverages routinely collected, clinically accessible data, making it readily translatable to real-world practice. Furthermore, the use of Extra Trees, a robust ensemble learning algorithm, and the comprehensive model evaluation using ROC curves, PR curves, calibration curves, and DCA, ensures the rigor and reliability of our findings. In addition to its superior performance metrics, the selection of the Extra Trees algorithm over other well-performing models such as Random Forest, Gradient Boosting, and Decision Tree was based on several key considerations. First, although Random Forest and Gradient Boosting also demonstrated strong predictive capacity, Extra Trees consistently achieved the highest AUC (0.97) and maintained robust sensitivity and specificity in both training and validation sets. Second, the Extra Trees algorithm, by introducing more randomness in the selection of cut-points and averaging across a large number of de-correlated trees, helps mitigate overfitting and improves generalizability – features that are particularly advantageous in datasets with limited sample sizes, such as ours. Third, we found that Extra Trees provided more stable and interpretable SHAP outputs for visualizing feature importance, which was crucial for model transparency and clinical translation. Nonetheless, we acknowledge that other tree-based models also showed favorable performance and could serve as complementary methods for external validation or ensemble approaches in future work. This will be discussed as part of our future research direction.

A key strength of our study is the visualization of feature importance using SHAP analysis. This not only enhances the interpretability of our “black box” ML model but also provides clinically relevant insights into the relative contribution of each predictor.^[35,36]^ Understanding which factors are most influential in driving the model’s predictions can improve clinician confidence in adopting and applying the model in practice.

Moreover, the development of a user-friendly web-based calculator is a significant step towards facilitating clinical implementation.^[37]^ By providing a readily accessible tool for risk prediction, we aim to bridge the gap between research and clinical practice, enabling clinicians to easily utilize our model to aid in decision-making for patients with GBPs. This calculator has the potential to streamline risk assessment, reduce reliance on subjective interpretation of ultrasound features, and ultimately contribute to more personalized and evidence-based management strategies.

Despite these strengths, our study has limitations. First, the retrospective, single-center design with a relatively small sample size (n = 202) may limit the generalizability of our findings to other populations and healthcare settings. Second, the model is based exclusively on structured clinical, laboratory, and ultrasound data; it does not incorporate more granular data types such as image-derived radiomics, genetic or molecular biomarkers, or intraoperative findings, which may enhance predictive performance in certain clinical scenarios. Third, while we aimed for model parsimony to ensure interpretability and clinical feasibility, this simplification may limit its adaptability to rare or atypical polyp presentations not well represented in the training data. For instance, polyps with unusual echogenic patterns, overlapping comorbidities (e.g., primary sclerosing cholangitis), or very small sample subsets (e.g., patients under 30 years old) might not be accurately predicted due to underrepresentation.

Furthermore, ML models inherently learn patterns from the data they are trained on; thus, this model may not generalize well to data collected from different healthcare systems, using different ultrasound machines, laboratory ranges, or clinical guidelines. Continuous model retraining or fine-tuning may be necessary in such contexts. In addition, although Extra Trees was selected for its interpretability and generalization, ensemble methods can be sensitive to imbalanced data distributions and may perform poorly when confronted with out-of-distribution cases or corrupted inputs.

To further refine the model, future iterations could explore the inclusion of multimodal data such as radiomics features extracted from imaging, deep learning-based interpretation of raw ultrasound data, and genomic or proteomic biomarkers. Incorporating patient-specific risk factors (e.g., family history, metabolic syndrome, or biliary tract disease) may also improve individualized risk prediction. Moreover, adaptive models that account for different regional clinical practices, laboratory ranges, and imaging protocols could enhance model portability across institutions. Finally, creating ensemble or hybrid frameworks that combine the strengths of multiple ML algorithms may boost robustness, especially in edge-case predictions.

Future research directions should focus on external validation of our model in diverse patient populations and clinical settings. Prospective studies are needed to evaluate the clinical impact of using our model to guide management decisions for patients with GBPs, specifically assessing its ability to reduce unnecessary cholecystectomies and improve patient outcomes. Furthermore, exploring the cost-effectiveness of implementing our model in routine clinical practice is essential for its widespread adoption. Investigating the potential of combining our model with other diagnostic modalities, such as advanced imaging or molecular markers, could further refine risk stratification strategies.

## 5. Conclusion

Our study demonstrates the successful development and validation of a noninvasive ML model for predicting gallbladder polyp malignancy, integrating ultrasound features and serological markers. The combined model, leveraging readily available clinical data and facilitated by a user-friendly web calculator, offers a promising tool to improve risk stratification and potentially optimize the management of patients with GBPs. Further validation and prospective clinical implementation studies are warranted to fully realize the clinical potential of this novel approach.

## Acknowledgments

This study received no external funding. The authors declare no conflicts of interest.

## Author contributions

**Conceptualization:** Yan Yang, Haibin Tu.

**Data curation:** Yan Yang, Haibin Tu.

**Formal analysis:** Yan Yang, Haibin Tu.

**Funding acquisition:** Yan Yang, Haibin Tu.

**Investigation:** Yan Yang, Haibin Tu, Youguo Lin.

**Methodology:** Yan Yang, Haibin Tu, Youguo Lin.

**Project administration:** Haibin Tu, Youguo Lin.

**Resources:** Haibin Tu, Youguo Lin, Jianting Wei.

**Software:** Youguo Lin, Jianting Wei.

**Supervision:** Youguo Lin, Jianting Wei.

**Validation:** Youguo Lin, Jianting Wei.

**Visualization:** Jianting Wei.

**Writing – original draft:** Jianting Wei.

**Writing – review & editing:** Jianting Wei.

## Supplementary Material


