# Supplementary material for: A machine learning model combining ultrasound features and serological markers predicts gallbladder polyp malignancy: A retrospective cohort study
Source: Medicine (Baltimore). 2025 Sep 12;104(37):e44371. doi: 10.1097/MD.0000000000044371 (PMC12440525; doi:10.1097/MD.0000000000044371)
Supplement: Supplementary file 1 [file medi-104-e44371-s001.docx]

| Supplymentary table 1 Information in training set | | | | | |
| --- | --- | --- | --- | --- | --- |
| Variable | Overall | Polyp | Cancer | Statistic | P_Value |
| female | 67 (47.2%) | 51 (47.7%) | 16 (45.7%) | 0 | 1 |
| male | 75 (52.8%) | 56 (52.3%) | 19 (54.3%) |  |  |
| stalk(sessile) | 85 (59.9%) | 75 (70.1%) | 10 (28.6%) | 17.23 | <0.01 |
| stalk(pedunculated) | 57 (40.1%) | 32 (29.9%) | 25 (71.4%) |  |  |
| Waist_Hip_Ratio(Normal) | 67 (47.2%) | 50 (46.7%) | 17 (48.6%) | 0 | 1 |
| Waist_Hip_Ratio(fatty) | 75 (52.8%) | 57 (53.3%) | 18 (51.4%) |  |  |
| Hypertension(no) | 104 (73.2%) | 79 (73.8%) | 25 (71.4%) | 0 | 0.95 |
| Hypertension(yes) | 38 (26.8%) | 28 (26.2%) | 10 (28.6%) |  |  |
| Diabetes(no) | 124 (87.3%) | 95 (88.8%) | 29 (82.9%) | 0.39 | 0.53 |
| Diabetes(yes) | 18 (12.7%) | 12 (11.2%) | 6 (17.1%) |  |  |
| Hyperlipidemia(no) | 115 (81%) | 87 (81.3%) | 28 (80%) | 0 | 1 |
| Hyperlipidemia(yes) | 27 (19%) | 20 (18.7%) | 7 (20%) |  |  |
| Alcohol history(no) | 134 (94.4%) | 104 (97.2%) | 30 (85.7%) | 4.56 | 0.03 |
| Alcohol history(yes) | 8 (5.6%) | 3 (2.8%) | 5 (14.3%) |  |  |
| Polyp_Location(bottom) | 54 (38%) | 34 (31.8%) | 20 (57.1%) | 8.46 | 0.01 |
| Polyp_Location(body) | 54 (38%) | 47 (43.9%) | 7 (20%) |  |  |
| Polyp_Location(neck) | 34 (23.9%) | 26 (24.3%) | 8 (22.9%) |  |  |
| Polyp_Number(single) | 68 (47.9%) | 51 (47.7%) | 17 (48.6%) | 0 | 1 |
| Polyp_Number(multiple) | 74 (52.1%) | 56 (52.3%) | 18 (51.4%) |  |  |
| Echo_Homogeneity | 52 (36.6%) | 43 (40.2%) | 9 (25.7%) | 1.8 | 0.18 |
| Echo_Heterogeneous | 90 (63.4%) | 64 (59.8%) | 26 (74.3%) |  |  |
| Polyp Surface Smoothness | 122 (85.9%) | 92 (86%) | 30 (85.7%) | 0 | 1 |
| Polyp Surface Coarse | 20 (14.1%) | 15 (14%) | 5 (14.3%) |  |  |
| Hyperechoic_Foci(no) | 77 (54.2%) | 52 (48.6%) | 25 (71.4%) | 4.66 | 0.03 |
| Hyperechoic_Foci(yes) | 65 (45.8%) | 55 (51.4%) | 10 (28.6%) |  |  |
| Gallstones(no) | 43 (30.3%) | 30 (28%) | 13 (37.1%) | 0.65 | 0.42 |
| Gallstones(yes) | 99 (69.7%) | 77 (72%) | 22 (62.9%) |  |  |
| Gallbladder_Smooth | 108 (76.1%) | 81 (75.7%) | 27 (77.1%) | 0 | 1 |
| Gallbladder_Roughness | 34 (23.9%) | 26 (24.3%) | 8 (22.9%) |  |  |
| Viral_Hepatitis(no) | 59 (41.5%) | 42 (39.3%) | 17 (48.6%) | 0.6 | 0.44 |
| Viral_Hepatitis(yes) | 83 (58.5%) | 65 (60.7%) | 18 (51.4%) |  |  |
| age(years) | 59.45 ± 15.95 | 57.22 ± 15.91 | 66.26 ± 14.26 | 1244 | 0.003 |
| diameter(mm) | 13.75 ± 3.43 | 12.76 ± 3 | 16.78 ± 2.84 | 632 | <0.001 |
| NLR | 3.03 ± 1.82 | 2.77 ± 1.79 | 3.82 ± 1.69 | 1262.5 | 0.004 |
| BMI | 22.1 ± 3.96 | 22.3 ± 4.02 | 21.49 ± 3.76 | 2144 | 0.2 |
| TBIL（mg/dL） | 16.72 ± 8.79 | 16.46 ± 7.91 | 17.51 ± 11.14 | 1924 | 0.809 |
| ADA（IU/L） | 12.58 ± 8.14 | 12.42 ± 8.3 | 13.06 ± 7.73 | 1741 | 0.535 |
| CEA（ng/mL) | 4.21 ± 3.96 | 4.09 ± 3.98 | 4.57 ± 3.9 | 1603.5 | 0.204 |
| CA199（U/mL） | 23.54 ± 19.9 | 22.8 ± 19.76 | 25.81 ± 20.47 | 1678.5 | 0.36 |
| ALP（IU/L） | 106.18 ± 52.89 | 105.38 ± 54.22 | 108.6 ± 49.27 | 1719 | 0.469 |
| AST（IU/L） | 28.88 ± 15.58 | 29.33 ± 14.86 | 27.52 ± 17.74 | 2214.5 | 0.106 |
| ALT（IU/L） | 33.48 ± 21.56 | 33.08 ± 21.46 | 34.72 ± 22.13 | 1791.5 | 0.703 |

NLR: Neutrophil-to-Lymphocyte Ratio; BMI: Body Mass Index; TBIL: Total Bilirubin; ADA: Adenosine Deaminase; CEA: Carcinoembryonic Antigen; CA199: Carbohydrate Antigen 19-9; ALP: Alkaline Phosphatase; AST: Aspartate Aminotransferase; ALT: Alanine Aminotransferase.
